# Predicting College Students’ Adoption of Technology for Self-Directed Learning: A Model Based on the Theory of Planned Behavior With Self-Evaluation as an Intermediate Variable

**DOI:** 10.3389/fpsyg.2022.865803

**Published:** 2022-05-09

**Authors:** Sy-Yi Tzeng, Kuen-Yi Lin, Chih-Yu Lee

**Affiliations:** ^1^Department of Graphic Communications and Digital Publishing, Shih Hsin University, Taipei City, Taiwan; ^2^Department of Technology Application and Human Resource Development, National Taiwan Normal University, Taipei City, Taiwan

**Keywords:** intention, intermediate effect, self-directed learning behaviors, self-evaluation, theory of planned behavior

## Abstract

Many studies assume a significant relationship between intention and behavior. However, the data do not always support this assumption. This study used a modified version of social cognitive theory with self-evaluations as an intermediate variable to explore and resolve the problems associated with applying the theory of planned behavior to explain students’ adoption of technology for self-directed learning. We surveyed 285 college students who enrolled in an e-book publishing course using multifaceted technological learning tools. We found that, as an intermediate variable, self-evaluation enhanced the influence of intentions on behavior and improved the accuracy of predictions of college students’ adoption of technology for self-directed learning. Students’ attitudes and perceived behavioral control were important factors influencing their adoption of technology for self-directed learning through their effects on intention; subjective norms were not important in this respect.

## Introduction

The application of new technologies, such as the Internet, social media, cloud computing, certain types of media, and mobile learning, has changed the face of education. Students have gradually transitioned from learners with external beliefs to internally motivated knowledge seekers. Researchers generally believe that the modern digital world, i.e., the Internet and social media, has encouraged self-directed learning behaviors. Researchers are increasingly applying social cognitive theory (SCT) to explore the use of technology by students (Lee, 2010; Chang et al., 2011, 2020; Lin, 2012; Lin and Wang, 2012; Cator and Adam, 2013; Dede and Grimson, 2013; Camilleri and Camilleri, 2021).

One of the fundamental principles of SCT is that learners wish to develop ways to control events (self-directed learning behavior; Bandura, 1977). In terms of the conceptual framework for learning, SCT emphasizes the interactions among learners’ perceived intrinsic motivation, learning behaviors, and learning environments. SCT principles have been extensively applied to self-regulation (Zimmerman, 2000, 2001), cognition-motivation-control (Shih, 2008; Chang et al., 2020; Camilleri and Camilleri, 2021), expectation-confirmation (Lee, 2010; Chang et al., 2011; Hung et al., 2011; Lin, 2012; Lin and Wang, 2012), and expectancy-value models of motivation (Jodl et al., 2001; Watt et al., 2012; van Tuijl and van der Molen, 2016). Based on self-evaluation data, SCT posits that self-directed learning behaviors result from an intrinsic evaluation of the self, including one’s behavior, as well as the environment. Self-evaluation is an important factor in students’ perceived intrinsic motivation to learn.

Many empirical studies on education employ Ajzen’s (1985, 1991) theory of planned behavior (TPB) to explain students’ adoption of technology (Liaw et al., 2007; Liaw, 2008; Shih, 2008; Sanchez-Franco et al., 2009; Cheon et al., 2012; Agudo-Peregrina et al., 2014). However, the TPB only explores behavioral attitudes, subjective norms, perceived behavioral control (PBC), intention, and behaviors. The TPB emphasizes extrinsic sources of control of an actor’s beliefs regarding behavior. The self-perceived intrinsic motivations highlighted in SCT (i.e., outcome expectations, satisfaction, values, goal setting, and goal progress) are lacking from the TPB; therefore, some researchers have integrated SCT to modify the theory (e.g., Liaw, 2008; Shih, 2008). Studies that use TPB are limited in their ability to explain self-directed learning behaviors.

We took an SCT perspective to overcome the shortcomings of using the TPB to explore students’ adoption of technology for self-directed learning. The TPB assumes that self-directed learning behaviors result from increasing or maintaining self-evaluation relationships. We used self-evaluations as intermediate variables to modify the TPB and thus strengthen the causal relationship between intention and behavior.

Our main research questions are as follows: (1) Can self-evaluations be used to modify the TPB to accurately predict the adoption of technology for self-directed learning? and (2) How do the various factors in the modified TPB model interact?

## Theoretical Background

### Investigation of the Adoption of Technology for Self-Directed Learning

Developments in information technology have driven interest in its application to research on self-directed learning behaviors. Technology has influenced the overall learning environment; starting with computer-assisted instruction, technology has evolved to encompass web-based learning, e-learning, and mobile learning, etc. Adopting technology for self-directed learning is operationally defined as using technology to enhance learning behaviors (Pellegrino and Hilton, 2012; Cator and Adam, 2013; Dede and Grimson, 2013). Students use technology for different types of learning, including e-learning, learning based on the application of tools for specific tasks (Lin and Wang, 2012), and learning pertaining to access logs (Agudo-Peregrina et al., 2014), examinations (Mohammadi, 2015), participation on social platforms (such as blogs or Facebook) (Zhang et al., 2012); sharing (Chen et al., 2009; Lai and Chen, 2011) and cloud computing for collaborative projects (e.g., Google applications) (Cheung and Vogel, 2013). Study management systems providing data on logins, downloads, uploads, pages viewed, and reactions are also used (Pynoo and Van Braak, 2014); moreover, technology can facilitate teaching (Motaghian et al., 2013; Chen et al., 2015).

Researchers from different eras have posited technological theories. Early education researchers focused on stimulus and response learning, for example by using artificial intelligence to provide students with personalized practice questions based on their strengths and weaknesses to improve knowledge and skills (Carbonell and Collins, 1973; Brown and Burton, 1978). Other researchers focused on the relationships of psychological factors with technology use to achieve learning goals. Several theoretical models, such as the technology acceptance model (Davis, 1986), were proposed that focused on the influence of personal attitudes toward technology. The influence of such attitudes and social norms have also been explored (Fishbein and Ajzen, 1975; Venkatesh and Davis, 2000). Moreover, many researchers explored the influence of environmental factors, such as opportunities, skills, conditions, and resources, as exemplified by the TPB (Ajzen, 1985, 1991), technology acceptance model 3 (Venkatesh and Bala, 2008), unified theory of acceptance and use of technology (Venkatesh et al., 2003), and extended unified theory of acceptance and use of technology (Venkatesh et al., 2012). Based on previous analyses, we believe that personal attitudes, subjective social norms, and personal beliefs are key factors in the utilization of technology for self-directed learning. The TPB (Ajzen, 1985, 1991) was adopted in this study as the basic theoretical model.

With the advent of the Internet, cloud computing, and social media, researchers have begun to apply models based on SCT to understand students’ motivations and adoption of technology for self-directed learning, such as the cognition-motivation-control model (Shih, 2008; Chang et al., 2020; Camilleri and Camilleri, 2021), expectation-confirmation model (Lee, 2010; Chang et al., 2011; Hung et al., 2011; Lin, 2012; Lin and Wang, 2012), and expectancy-value model of motivation (Jodl et al., 2001; Watt et al., 2012; van Tuijl and van der Molen, 2016). Educational technology is not as limited as before due to the emergence of personal desktop computers and unlimited Internet access. Education technology has transformed learning into a more self-directed process, facilitated by the wide variety of devices and operating systems, and opportunities for collaborative learning. Against this background, research’ interest is moving from extrinsic to intrinsic motivation. The intrinsic motivation factors according to SCT are gaining more attention, and some studies have attempted to combine SCT with the TPB (Shih, 2008). This study expands on previous SCT researchers’ ideas to reevaluate students’ perceived intrinsic motivation (i.e., self-evaluation) and technology adoption for self-directed learning.

### Role of Self-Evaluation in the Theory of Planned Behavior

According to Ajzen (1985, 1991), intention affects behavior. The TPB holds that beliefs determine an actor’s intentions, which are based on attitudes, subjective norms, and PBC. Although some research supports the assumptions of the TPB (Lin, 2012; Cheung and Vogel, 2013; Motaghian et al., 2013; Chen et al., 2015; Mohammadi, 2015; Chu and Chen, 2016), other studies did not show a link between intention and behavior (Liaw, 2008; Agudo-Peregrina et al., 2014; Pynoo and Van Braak, 2014). Pynoo and Van Braak (2014) call this the “intention-behavior gap.” In Taiwan, Liaw (2008) studied the use of the blackboard e-learning system for college students, and found that intention was not significantly correlated with the students’ self-reported study behaviors. This may be because the technology did not increase the effectiveness of e-learning. Agudo-Peregrina et al. (2014) compared learning behavior associated with the adoption of e-learning technology between graduates and lifelong learners in Spain, and found that only lifelong learners’ intention had a significant effect on behavior. They concluded that this was due to lifelong learners’ intrinsic motivation to enroll in online courses, to access new opportunities in the labor market. However, much research ignores the relationship between intention and behavior entirely (Tao et al., 2009; Lee, 2010; Liu et al., 2010; Teo and Lee, 2010; Hung et al., 2011; Cheon et al., 2012; Lin and Wang, 2012). This may be because standard surveys simply do not reveal this relationship, so researchers limit their models to take account of intention only. Therefore, we excluded extrinsic motivation from our analyses, and instead focused on the influence of students’ intrinsic motivation.

In TPB Ajzen’s (1985, 1991), behavioral factors are composed of several beliefs. However, they are often mistaken for intrinsic motivation because beliefs, attitudes, norms, and PBC depend on the environment. Intention is a reasonable concept to explain consumer behavior when applying the TPB; when the environment is favorable, consumers will consume. However, when the TPB is used to analyze students’ adoption of technology for self-directed learning, although there may be a positive intention to adopt technology for learning due to a lack of intrinsic motivation, this may not translate into actual adoption of technology for self-directed learning.

Based on self-evaluation data, SCT holds that self-directed learning behaviors result from an intrinsic evaluation of the self, including one’s behavior, as well as the environment (Bandura, 1986). Schunk (2020) further emphasizes that self-evaluation, involving self-judgments of current performance based on the progress toward the current goal, is an important factor in a student’s intrinsic motivation. From the perspective of SCT, self-evaluation is based on outcome expectations, satisfaction, values, and goal setting and progress. For example, Shih’s (2008) research based on a cognition-motivation-control perspective suggests that personal outcome expectations are a form of behavioral motivation. Personal outcome expectations significantly influence the intention to use the Internet for academic learning.

Using the expectation-confirmation model, many researchers have shown that satisfaction with a learning management system can influence behavioral performance and intention (Lee, 2010; Chang et al., 2011; Hung et al., 2011; Lin, 2012; Lin and Wang, 2012). Lin (2012) suggested that students may not adopt technology if they perceive it as an unimportant for the learning process, do not understand the material to be learned, cannot easily achieve their goals during the study process, or believe that technology is not easy to use, etc.

The expectancy-value model of motivation focuses on beliefs regarding one’s own ability, referred to as success expectancies or self-concept (Jodl et al., 2001; Watt et al., 2012; van Tuijl and van der Molen, 2016), where the actors consider the following: “Can the behavior be performed successfully?” and “Can the technology help me learn more efficiently?” In the expectancy-value model of motivation, self-evaluation describes beliefs regarding one’s ability to adopt technology for self-directed learning, referred to as success expectancies (Watt et al., 2012) or self-concept (Jodl et al., 2001), as stated above. These definitions are identical to that for self-efficacy (van Tuijl and van der Molen, 2016); all of these concepts of self-evaluation help predict whether a student will adopt a given technology. We speculate that students’ self-evaluations of outcome expectations, values, and goal setting and progress will influence the relationship between intention and behavior.

Self-evaluation promotes understanding of the relationship between intention and self-directed learning behaviors. Low self-esteem and negative self-perceptions will not necessarily diminish motivation if students believe they can succeed, even if their current approach is ineffective (Bandura, 1986). Such students may work harder, persist longer, or adopt what they believe is a better strategy (Schunk, 2020). Schunk (1991, 2020) stated that positive self-evaluations can lead students to believe that studying is effective, such that they work diligently because they believe that they have the intrinsic ability to improve. Students with more positive self-evaluations exhibit more effective and multifaceted self-directed learning behaviors. However, negative self-evaluations may render students unwilling to persist with learning (Liaw, 2008; Lin, 2012; Agudo-Peregrina et al., 2014), because they believe that their abilities or strategies are insufficient. Therefore, positive self-evaluations will promote the adoption of a particular technology to aid learning. On the other hand, students with a positive intention but negative self-evaluation (perhaps due to previous failures or a lack of learning efficiency) will not demonstrate self-regulatory behaviors. This helps explain why students with positive intentions can lack self-directed learning behaviors, and may only use technology if forced to by a teacher. Against this background, we propose the following hypotheses:

H1. Learners with more positive intentions will also have more positive self-evaluations.
H2. Learners with more positive self-evaluations will adopt technology to aid multifaceted self-directed learning behaviors.

### Antecedents of the Theory of Planned Behavior

Attitude is defined herein as students’ perceptions of technology for learning. Fishbein and Ajzen (2010) proposed that the attitude toward a behavior is an essential aspect of learners’ intentions. Studies have empirically proved the significance of learners’ attitudes toward the intention to adopt technology (Chen et al., 2009; Lee, 2010; Teo and Lee, 2010; Cheung and Vogel, 2013). Accordingly, we propose hypothesis (H3), as follows:

H3. A learner with a more positive attitude will have a greater intention to adopt technology.

Subjective norms are defined herein as the influence of instructors and peers on students’ perceptions of technology adopted for learning. Fishbein and Ajzen (2010) proposed that subjective norms were important factors affecting intention. However, in some studies, subjective norms had a non-significant effect on intention (Lee et al., 2011; Cheung and Vogel, 2013). Lee et al. (2011) stated that subjective norms significantly affect behavioral intention. For example, students who use a platform that integrates text, images, and audio (e.g., Google or YouTube) can interact online through email, use electronic bulletin boards, and take online quizzes. New users of a technology can learn to use it easily if helped by others; however, over time, the intention to continue using a technology depends mainly on personal motivation. Nevertheless, other studies have reported a significant positive relationship between subjective norms and intention (Lee, 2010; Cheon et al., 2012; Chu and Chen, 2016). Thus, we propose another (tentative) hypothesis (H4), as follows.

H4. A learner with more positive subjective norms will have a greater intention to adopt technology.

Perceived behavioral control is operationally defined herein as students’ beliefs about their degree of control over the technology to be adopted to aid the learning process. These beliefs may be informed by whether they have used the technology before and, if so, how often. PBC directly affects intention and behaviors (Fishbein and Ajzen, 2010). Students’ beliefs (e.g., regarding opportunities, resources, and the environment) are key elements influencing their intention to adopt technology for learning purposes. Notably, Chen et al. (2009) showed that students’ extrinsic and intrinsic PBC are both significantly correlated with behavior, but only extrinsic PBC is significantly correlated with intention. Although they reported that PBC had a significant effect on intention, Sawang et al. (2014) suggested otherwise. Because these findings conflict with H4, further clarification is needed. Thus, we propose two more tentative hypotheses (H5 and H6), as follows:

H5. A learner with a higher PBC will have a greater intention to adopt technology.
H6. A learner with a higher PBC will adopt technology for learning in a multifaceted way.

Intention is operationally defined herein as the intention of the learner to adopt a particular technology. According to Ajzen (1991) and Fishbein and Ajzen (2010), student behavior may be directly influenced by behavioral intention. Although previous studies showed that a positive intention does not have a significant positive effect on learning behaviors (Liaw, 2008; Lin, 2012; Agudo-Peregrina et al., 2014), some research supports the assumptions of the TPB (Pynoo et al., 2011; Lin, 2012; Cheung and Vogel, 2013; Motaghian et al., 2013; Chen et al., 2015; Mohammadi, 2015; Chu and Chen, 2016). Thus, we propose another tentative hypothesis (H7):

H7. A learner with higher intention will adopt technology and show multifaceted self-directed learning behaviors.

## Research Methodology

### Research Framework and Hypotheses

**FIGURE 1:**
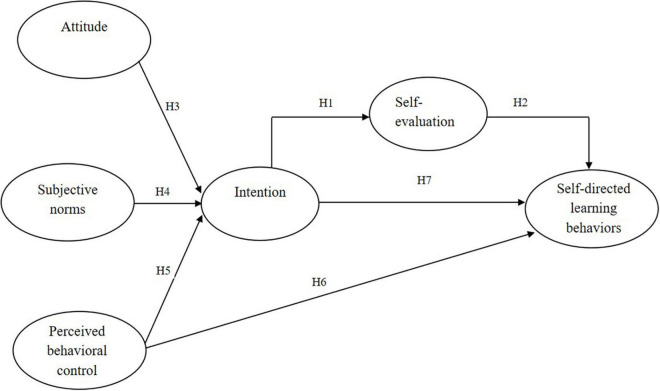
Research framework and hypotheses.

Our model based on the TPB included self-evaluation as an intermediate variable. Conceiving of self-evaluation as a combination of perceived intrinsic motivations, as in SCT, should help us understand students’ adoption of technology for self-directed learning. Figure 1 depicts our research model, which extends Ajzen’s TPB by adding one intermediate variable (self-evaluation) between intention and self-directed learning behaviors.

### Variables and Questionnaire Items

The final questionnaire used in this study consisted of 27 items to assess the 6 constructs of the proposed research model. The items in the questionnaire were scored using a 7-point Likert scale ranging from 1 (strongly disagree) to 7 (strongly agree).

#### The Adoption of Technology for Self-Directed Learning

This research uses subjective measures, as recommended by Ajzen (2019), and the binary choice technique to assess the extent of use of technology for self-directed learning according to the number of study behaviors exhibited that involve technology (Lai and Chen, 2011; Schunk, 2020); the more of those behaviors that the students demonstrate, the greater the degree of adoption of technology for self-directed learning. To this end, questionnaire items such as “When doing homework, do I use technological tools,” “When producing graphics, I use technological tools,” “When publishing articles, I use technological tools,” “During discussions with team members, I use technological tools,” “When working on an e-book project, I use technological tools,” and “When participating in classroom competitions, do use technological tools” were included. The technological tools of interest included search engines (e.g., Google), video platforms (e.g., YouTube), social media platforms (e.g., Facebook, Line, and Instagram), and cloud computing for collaborative projects (e.g., Google Docs).

Behaviors are usually assessed based on objective (e.g., data from systematic records) or subjective (e.g., personal accounts) measures (Fishbein and Ajzen, 2010). As objective measurement of behaviors can be difficult (Fishbein and Ajzen, 2010), past research mainly used subjective self-report data, including assessment scales (Ngai et al., 2007; Wang and Wang, 2009; Chen, 2010; Cheung and Vogel, 2013; Chen et al., 2015), frequency data (for a given behavior) (Pynoo et al., 2011; Pynoo and Van Braak, 2014; Ajzen, 2019), reports of the time spent engaging in a particular behavior (Pynoo et al., 2011; Chu and Chen, 2016), and simple binary assessments (use/non-use of a specific approach) (Lai and Chen, 2011). It is important to emphasize that using technology for learning is a multifaceted process, and to avoid missing data when using self-report surveys of students’ adoption of technology for self-directed learning. A binary analysis approach is preferable to avoid differences between reported and actual behavior. Therefore, this study adopted Lai and Chen’s (2011) binary choice technique.

#### Self-Evaluation

Self-evaluation questions were developed based on the studies of Jodl et al. (2001), Shih (2008), Lin (2012), and Watt et al. (2012). Notably, previous studies utilized self-evaluation questions related to expected consequences that were in the future tense (e.g., “will help,” “will improve,” etc.); however, the responses were not associated with behavior (McGill and Klobas, 2009). Thus, we did not use the future tense. Self-evaluation is defined herein as self-regulation of the adoption of technology by the learner. We were interested in the use of technology to solve academic problems encountered during their studies (outcome expectations) (Shih, 2008), as well as in successful self-initiated application of technology to study for major courses in an individualized manner (confirmation) (Lin, 2012), technology-aided goal setting and progress (Watt et al., 2012), and enhancement of knowledge and skills in the major field of study through the use of technology (values) (Jodl et al., 2001). Self-evaluation was assessed via questionnaire items such as “I always use technology on my own to solve problems I encounter during coursework,” “I have my own methods of using technology to help with my major courses,” “I can control the pace of my learning by using technology,” and “I am very accustomed to using technology to improve my knowledge and skills.”

#### Intention

Intention-related questions were developed based on Ajzen (1991) and Fishbein and Ajzen (2010), including “I always use technology to help me study for my major courses,” “If it is permitted, I will use technology to the maximum extent possible in my major courses,” “I will use technology in the future to obtain information related to my major,” and “I am happy to recommend technology for cooperative study activities.”

#### Attitude

Attitude-related questions were similarly developed based on Ajzen (1991) and Fishbein and Ajzen (2019), including “It is fun to use technology to do my coursework,” “In my major classes, it is smart to use technology to learn,” “I enjoy using technology as I study for my major courses,” and “Technology promotes a self-directed study environment.”

#### Subjective Norms

Subjective norm-related questions were again developed based on Ajzen (1991) and Fishbein and Ajzen (2010), including “If the majority of my friends and partner used technology to study, I would use it, too,” “I adopt technology when respected teachers or friends recommend it to me for my studies,” “The opinions of the important people around me affect my decision to use technology as a study tool,” and “If my friends and partner all used technology to communicate, I would use it, too.”

#### Perceived Behavioral Control

Perceived behavioral control-related questions were also developed based on Ajzen (1991) and Fishbein and Ajzen (2010), including “During my studies, I often use technology to enhance my learning abilities,” “I often use technology to help me complete creative projects,” “When I have problems with my coursework, I often use technology to solve them,” and “I am always trying new technologies to see if they can help me academically.”

### Sampling

The students enrolled in this study were from two universities in northern Taiwan; a theoretical sampling approach was used. They were taking courses related to new media technology, such that they had to use technology for their coursework including mobile devices, the Internet, and cloud computing (smartphones, tablets, notebook computers, etc.). Education was provided face-to-face, i.e., in the classroom, as well as via the Internet, iOS and Android mobile devices. The students participated in virtual interactive activities with teachers and peers, through social media platforms (e.g., online peer discussions, collaborative group work, and online question and answer sessions with instructors). All of participants had enrolled in an e-book publishing course involving multifaceted technological learning tools. Our paper questionnaire survey was completed between weeks 13 and 15 of the semester, which lasted for 18 weeks. A total of 360 completed questionnaires s were collected. After discarding 75 incomplete questionnaires, the remaining 285 were further analyzed.

### Reliability and Validity of the Questionnaire

The original questionnaire was in English but was translated into Chinese for this study. One native English speaker verified that the Chinese version of the questionnaire was valid, based on suggestion Ary et al. (2018). The questionnaire content was also validated in a pilot study by an academic expert, e-learning specialist, learning theory specialist, and 30 students reporting technology-enhanced learning experiences. The subjects of the pilot study were college students who participated in the abovementioned courses. The 30 completed questionnaires were assessed for skewness, kurtosis, and variance. Analysis of individual survey items was conducted. Items that did not reach significance (average scores >6 or <2) were omitted based on the results of confirmatory factor analysis (10 items in total).

Regarding instrument reliability, Cronbach’s alpha values should be >0.7 (Hair et al., 1998) and the average variance extracted (AVE) value should be >0.5 (Fornell and Larcker, 1981). All of our constructs had a Cronbach’s alpha value >0.7 (range: 0.80–0.91). The AVE values for the latent constructs were all >0.5 (range: 0.51–0.70). In addition, the square root of the AVE value was larger than the correlation coefficient, indicating satisfactory discriminant validity (Chin and Newsted, 1999).

## Results

### Subjects

All 285 subjects were undergraduate students (79.3% female). Most subjects were third- or fourth-year students (97.9%). In total, 70.9% of the students went online at any time, 23.2% went online at night, 4.6% went online during the day, and only 0.4% did not go online often. On encountering academic problems, the technology tools of choice were as follows: 97.2% of students used the Google search engine, 54.7% used YouTube, 41.1% used Facebook, and 31.2% used Line. The technological platforms and tools were used for homework (93.0% of students), e-book project work (81.1% of students), publishing tasks (61.4% of students), classroom competitions (56.8% of students), and discussions with team members (50.2% of students). These results indicated that the respondents were primarily users of knowledge-based, as opposed to social, technology. Search engines were generally viewed as beneficial for self-directed learning, indicating that knowledge-based technologies were widely accepted as study tools by the respondents.

### Model Fit

We used structural equation modeling (SEM) to test our research models and hypotheses. Due to the large number of samples (>200), we used the Bollen–Stine bootstrap *p*-value (Bollen and Stine, 1992). The TPB model with self-evaluation as an intermediate variable had an χ^2^/df < 3 (1.31), indicating a good data fit. This was supported by the other indices [comparative fit index (CFI) = 0.98, goodness of fit index (GFI) = 0.94, adjusted goodness of fit index (AGFI) = 0.91, standardized root mean square residual (SRMR) = 0.05, root mean square error of approximation (RMSEA) = 0.05]. For the TPB model, the χ^2^/df was <3 (1.55), again suggesting a good data fit. This was supported by the other indices (CFI = 0.97, GFI = 0.93, AGFI = 0.91, SRMR = 0.04, and RMSEA = 0.03) (Table 1).

**TABLE 1:** Goodness-of-fit indices of the models.

Goodness-of-fit indices	Criteria	TPB model with self-evaluation as an intermediary	TPB model
**χ^2^-statistic**	**Non-significant**	*Non-significant	*Non-significant

χ^2^/df	<3	1.31	1.55
CFI	>0.95	0.98	0.97
GFI	>0.90	0.94	0.93
AGFI	>0.80	0.91	0.91
SRMR	<0.08	0.05	0.04
RMSEA	<0.10	0.03	0.03

*Bollen–Stine-corrected p-value.

### Structural Equation Modeling

**FIGURE 2:**
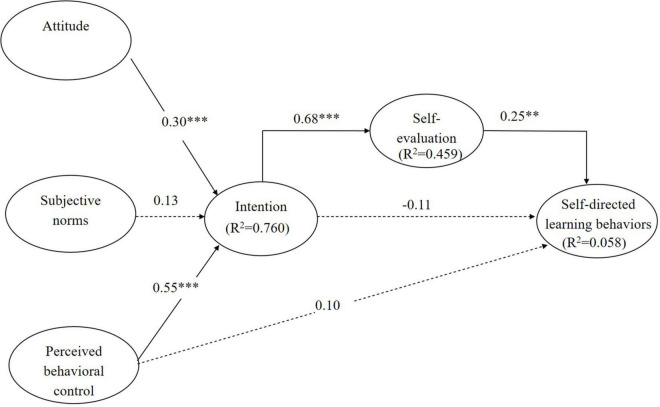
Path analysis of the TPB model including self-evaluation as an intermediate variable.

**FIGURE 3:**
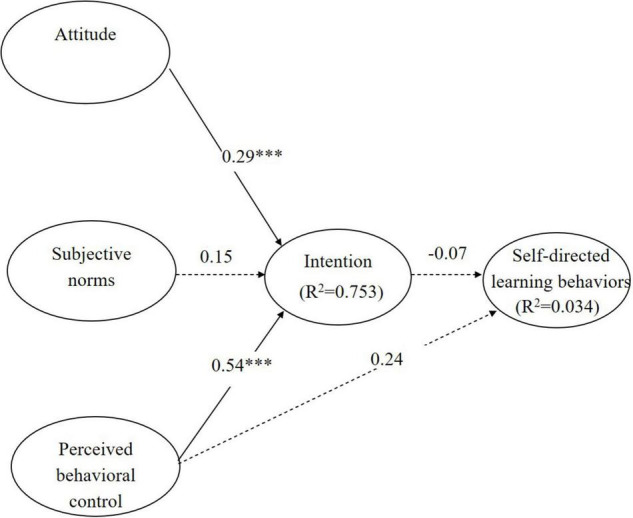
Path analysis of the model based on the original TPB.

Structural equation modeling was used to test the research hypotheses. Figures 2, 3 show all of the path coefficients, and the variance explained by the model with self-evaluation as an intermediate variable, and the model based on the original TPB.

#### Model With Self-Evaluation as an Intermediate Variable

Self-evaluation significantly mediated the relationships between intention and self-evaluation (β = 0.68, *p* < 0.001), and self-evaluation and motivation to learn (β = 0.25, *p* < 0.01) (Figure 2), which supports H1 and H2. Regarding antecedents, in the model with self-evaluation as an intermediate variable, attitude (β = 0.30, *p* < 0.001) and PBC (β = 0.55, *p* < 0.001) had positive effects on intention, thereby supporting H3 and H5. However, subjective norms (β = 0.13, *p* > 0.05) did not significantly affect intention; thus, H4 was not supported. Moreover, PBC and intention did not have significant effects on self-directed learning behaviors in either model (β = 0.10, *p* > 0.05; β = −0.11, *p* > 0.05); thus, H6 and H7 were not supported.

#### Model Based on the Original Theory of Planned Behavior

In the model based on the original TPB, there was a non-significant relationship between intention and behavior. The direct path from intention to self-directed learning behaviors was non-significant (β = −0.07, *p* > 0.05) (Figure 3), which does not support H7. Regarding antecedents, attitude (β = 0.29, *p* < 0.001) and PBC (β = 0.54, *p* < 0.001) had positive relationships with intention, supporting H3 and H5. However, subjective norms (β = 0.15, *p* > 0.05) did not significantly affect intention; thus, H4 was not supported. Moreover, PBC did not significantly affect self-directed learning behaviors (β = 0.24, *p* > 0.05); thus, H6 was not supported.

When self-evaluation was included as an intermediate variable (Figure 2), the influence of PBC on self-directed learning behaviors (β = 0.095, *p* > 0.05) was weaker than when self-evaluation was not an intermediate variable (β = 0.239, *p* > 0.05) (Figure 3).

## Discussion

This study examined the adoption of technology for self-directed learning by extending the TPB through inclusion of self-evaluation as an intermediate variable. We also explored the self-regulation concept of SCT as it pertains to the adoption of technology by students. Three noteworthy results were found and are discussed below.

### Theory of Planned Behavior Model With Self-Evaluation as an Intermediate Variable

Self-evaluation was a significant intermediate variable between intention and actual adoption of technology (see Figure 2); this finding accords with Bandura (1977, 1986, 1991) and Schunk (2020). Self-evaluation is an important factor in student motivation. Students may strongly believe that using technology can help them develop their values. When students believe that their studies are meaningful and they maintain a positive intention toward the use of technology, learning behaviors will naturally follow.

The findings regarding the intention to adopt technology for self-directed learning in this study echo previous research (Liaw, 2008; Agudo-Peregrina et al., 2014; Pynoo and Van Braak, 2014; see Figures 2, 3). However, the findings do not agree with Pynoo and Van Braak (2014), who believe that the gap between intention and behavior is mediated by subjective norms. Our findings suggest that intrinsic motivation (self-evaluation) rather than extrinsic motivation (intention) drives the adoption of technology for self-directed learning (Bandura, 1986).

### Relationships Among the Various Factors Included in the Models

Individual attitudes and PBC were the most important variables determining the intention to adopt technology (see Figures 2, 3), in line with most previous research (Chen et al., 2009; Fishbein and Ajzen, 2010; Teo and Lee, 2010; Cheung and Vogel, 2013). However, subjective norms did not show a significant relationship with intention, in contrast to Lee (2010), Cheon et al. (2012), and Chu and Chen (2016). In those studies, the relationship between intention and extrinsic belief was mainly mediated by subjective norms. This discrepancy may be explained by the participants in this study being third- and fourth-year students, who were typically long-time technology users.

The lack of an effect of subjective norms on intention described above also accords with Lee et al. (2011). Text-, image-, and video-based platforms, such as Google and YouTube, enable communication with other students via email, electronic bulletin boards, and online quizzes, while closed networks do not provide opportunities for learning involving other students. The majority of the students also reported being online most of the time, and used Google to solve academic problems (i.e., believed that search engines facilitate self-directed learning). As the students’ use of technology was relatively advanced, their study habits were not easily influenced by others.

The participants used technology to resolve academic problems and believed that technology was helpful for self-directed learning. Thus, social media platforms such as Facebook and Line were used less than Google and YouTube, and the influence of subjective norms on technology adoption for self-directed learning was weak.

A significant relationship between PBC and behavioral intention was found in this study, unlike that of Sawang et al. (2014). However, they mainly focused on social influences on student’s behavior in the context of a collectivist society. In our study, we assumed that the students were free to adopt technologies of their choice to aid their studies, and were not resource-limited. Accordingly, PBC exerted a significant influence on intention.

Finally, PBC did not directly affect behavior (see Figures 2, 3), especially in the model in which self-evaluation was an intermediate variable. In contrast to Chen et al. (2009), who reported that extrinsic beliefs about technology (PBC) and perceived intrinsic motivation to articulate ideas via technology (self-evaluation) significantly influenced behavior, we found that only self-evaluation had a significant effect on behavior. Although Fishbein and Ajzen’s (2010) theory posits that PBC has a significant direct effect on behavior, we found while outcome expectations, values, goal-setting, and self-evaluations of progress toward goals directly affected behavior, PBC did not promote or impede the adoption of technology. In addition, students’ perceptions of themselves were more important than PBC, i.e., self-evaluations regarding the adoption of technology were more important to self-directed learning than PBC.

## Conclusion

Self-evaluation involves comprehensive assessment of one’s abilities. Educators can improve students’ motivation to study using self-evaluation strategies. When students become frustrated, self-evaluations change, as should the methods and strategies used to resolve difficulties. Students with negative self-evaluations may feel frustrated because they lack expectations, values, or goals. Therefore, enhancing students’ self-perceived of success could be a target for further studies. Students with favorable self-evaluations are confident that they will succeed, even when they experience setbacks, and understand that it is only their approach that needs to change to achieve the desired outcome. To motivate students to keep studying, educators should set high goals for their students.

In the future, we recommend that the model in this study be applied only to groups of students studying the same major, because the use of technology may be very different among majors and departments. Similarly, we do not recommend that student data from different fields of study be combined. Our questionnaire survey took place during the semester to avoid any influence of final grades. However, the students could have been influenced by other courses or club activities; therefore, how best to adopt technology for self-directed learning requires further exploration.

There were some limitations to this study. For example, the study habits of students in different fields of study may not accord with the learning behaviors of interest in this study. Thus, the survey question items should be adjusted according to the likely applications of technology for specific subjects. The learning behaviors in this study were most relevant to design and communication students. Moreover, regarding SEM, the χ^2^ value may be too large if there more than 200 samples, as in this study. Bollen–Stine-corrected *p*-values are recommended in such cases, as applied herein.

Future research should further explore learning behavior based on self-evaluations, and assess the limitations of such methods. For example, when used with in the context of the TPB, self-evaluations are only applicable to self-directed learning behaviors (i.e., not to consumer behavior or technology adoption for non-learning purposes). Also, only older students should be used as study subjects because the self-regulated process of self-evaluation requires the ability to make accurate self-judgments; this ability may not be fully developed in youngers students. The results of the final pathway model in this study pertained to learning behaviors involving technology that were already quite prevalent among the students, and did not require contact with other students. We did not cover closed social networks for teachers and students, as these campus-specific social and mobile learning technologies are not popular; the inclusion of such networks might have led to different results.

## Data Availability Statement

The datasets presented in this study can be found in online repositories. The names of the repository/repositories and accession number(s) can be found in the link below: http://dx.doi.org/10.13140/RG.2.2.19004.72328.

## Author Contributions

S-YT was responsible for research design, conducting questionnaire survey, analyzing the data, and revised the manuscript (contributions to this research: 50%). K-YL was responsible for research design, collecting and analyzing the related literature, conducting questionnaire survey, and revised the manuscript (contributions to this research: 30%). C-YL was responsible for collecting and analyzing the related literature, and writing the manuscript (contributions to this research: 20%). All authors contributed to the article and approved the submitted version.

## Conflict of Interest

The authors declare that the research was conducted in the absence of any commercial or financial relationships that could be construed as a potential conflict of interest.

## Publisher’s Note

All claims expressed in this article are solely those of the authors and do not necessarily represent those of their affiliated organizations, or those of the publisher, the editors and the reviewers. Any product that may be evaluated in this article, or claim that may be made by its manufacturer, is not guaranteed or endorsed by the publisher.
